# Comparisons of brucellosis between human and veterinary medicine

**DOI:** 10.1080/20008686.2018.1500846

**Published:** 2018-07-24

**Authors:** Noah C. Hull, Brant A. Schumaker

**Affiliations:** Department of Veterinary Sciences, University of Wyoming, Laramie, Wyoming, USA

**Keywords:** Risk factors, pathogenesis, vaccines, diagnostics, infectious disease epidemiology, neglected disease

## Abstract

Brucellosis is the world’s most widespread zoonosis, but also ranks as one of the seven most neglected diseases, according to the World Health Organization. Additionally, it is recognized as the world’s most common laboratory-acquired infection. There are a reported 500,000 incident cases of human brucellosis per year. However, true incidence is estimated to be 5,000,000 to 12,500,000 cases annually. Once diagnosed, focus is directed at treating individual patients with antibiotic regimes, yet overall neglecting the animal reservoir of disease. Countries with the highest incidence of human brucellosis are Syria (1,603.4 cases per 1,000,000 individuals), Mongolia (391.0), and Tajikistan (211.9). Surveillance on animal populations is lacking in many developed and developing countries. According to the World Animal Health Information Database, Mexico had the largest number of reported outbreaks, 5,514 in 2014. Mexico is followed by China (2,138), Greece (1,268), and Brazil (1,142). The majority of these outbreaks is *Brucella abortus*, the etiologic agent of bovine brucellosis. Brucellosis is an ancient disease that still plagues the world. There are still knowledge gaps and a need for better diagnostics and vaccines to make inroads towards control and eradication.

## Introduction

*Brucella spp*., the etiologic agents of brucellosis, are Gram-negative, non-motile, facultative intracellular coccobacilli that can infect a wide range of mammalian species, including humans, and some amphibians [1,2]. There are 12 named *Brucella* spp., and four unnamed isolates (Table 1). *Brucella spp*. can be traced back 2.8 million years by presumptive evidence of pathologic changes in a late Pliocene hominin skeleton [3]. Additionally, molecular tests demonstrated the presence of *B. melitensis* DNA in a 700-year-old skeleton from medieval Italy [4]. The first description of the causative agent of brucellosis was made by Sir David Bruce in 1887 from the liver of a deceased solider on the island of Malta [5]. It was then termed *Micrococcus melitensis* [5]. Ten-years later, Bernard Bang isolated *Bacillus abortus* [6]. In honoring Sir Bruce, genus-nomenclature was standardized to *Brucella melitensis* and *Brucella abortus*, respectively [7]. Clinical human and animal brucellosis carries a plethora of synonyms including: undulant fever, Malta fever, Mediterranean fever, contagious abortion, Bang’s disease, Neapolitan fever, Crimean fever, and Corps disease [8]. A majority of these names are still used in varying parts of the world.

**Table 1.** *Brucella* species by host. Zoonotic potential is classified as pathogenicity and virulence in human hosts. Original citation indicates the original publication where the species was characterized.

Species	Natural host	Zoonotic Potential [8]	Original Citation
*B. melitensis*	Sheep, goats, and camels	Yes – High	[5]
*B. abortus*	Cattle, elk, and bison	Yes – High	[6]
*B. suis*	Pigs, hare, reindeer/caribou	Yes – High	[122]
*B. canis*	Dogs (domestic and wild)	Yes – Moderate	[123]
*B. ovis*	Sheep	No reported infections	[124]
*B. neotomae*	Desert wood rats	No reported infections	[125]
*B. ceti*	Cetaceans	Yes – Low	[126]
*B. pinnipedialis*	Pinnipeds	Yes – Low	
*B. microti*	Red foxes and common voles	No reported infections	[127]
*B. inopinata*	Unknown	Yes – High	[2,128]
*B. papionis*	Non-Human Primates	No reported infections	[129,130]
*B. vulpis*	Red fox	No reported infections	[131,132]
*Brucella NFXXXX*	Australian rat	No reported infections	[133,134]
*B. unnamed*	Blue dotted ray	No reported infections	[131]
*B. inopinata-like* *09RB8471*	African bullfrogs and Big-eyed tree frog	No reported infections	[2,135]
*Brucella UK8/14*	White’s tree frog	No reported infections	[136]

Brucellosis is the world’s most widespread zoonosis, but ranks as one of the seven most neglected diseases, according to the World Health Organization (WHO) [9,10]. There are approximately 500,000 reported incident cases of human brucellosis annually; however, true incidence is estimated at 5,000,000 to 12,500,000 cases annually [11–13].

Brucellosis is recognized as the world’s most common laboratory-acquired infection [14]. This is attributed to the low infectious dose, estimated between 10–100 bacterial cells by aerosol or subcutaneous route [15,16]. In the developing world, *B. abortus, B. melitensis* and *B. suis* are leading causes of animal and human brucellosis [17]. However, with the recent identification of novel strains of brucellae, the complete picture of animal and human health is still unknown. The geographical distribution is changing with brucellosis re-emerging in some areas. Consistent case-reports of animal and human brucellosis originate from all continents with exception of Antarctica, in which only animals have tested positive [9,18,19].

Although brucellosis is the most widespread zoonosis worldwide, it remains severely neglected as a potential cause for chronic, debilitating maladies, due to its non-descript clinical presentation in human populations. This leads to major economic ramifications due to the loss of normal daily activities [9]. Diagnoses are challenging in areas with endemic malaria due to wide ranging clinical presentations [20]. Once diagnosed, focus is directed at treating individual patients with antibiotic regimens, yet overall neglecting the animal reservoir of disease. A cornerstone of zoonotic infectious disease epidemiology is the One Health concept. The goal of One Health is to employ a multidisciplinary approach to achieve the best health for people, animals, and the environment [21]. The control or eradication of the disease in wildlife and agricultural animals is a prerequisite for the control of the disease in human populations [22].

The aim of this review is to provide updated information on the global presence of disease, describe pathogenesis, risk factors, and clinical presentation in both humans and animals, describe potential control strategies, and to outline current and forthcoming diagnostics.

## Global presence of disease

**Figure 1.**
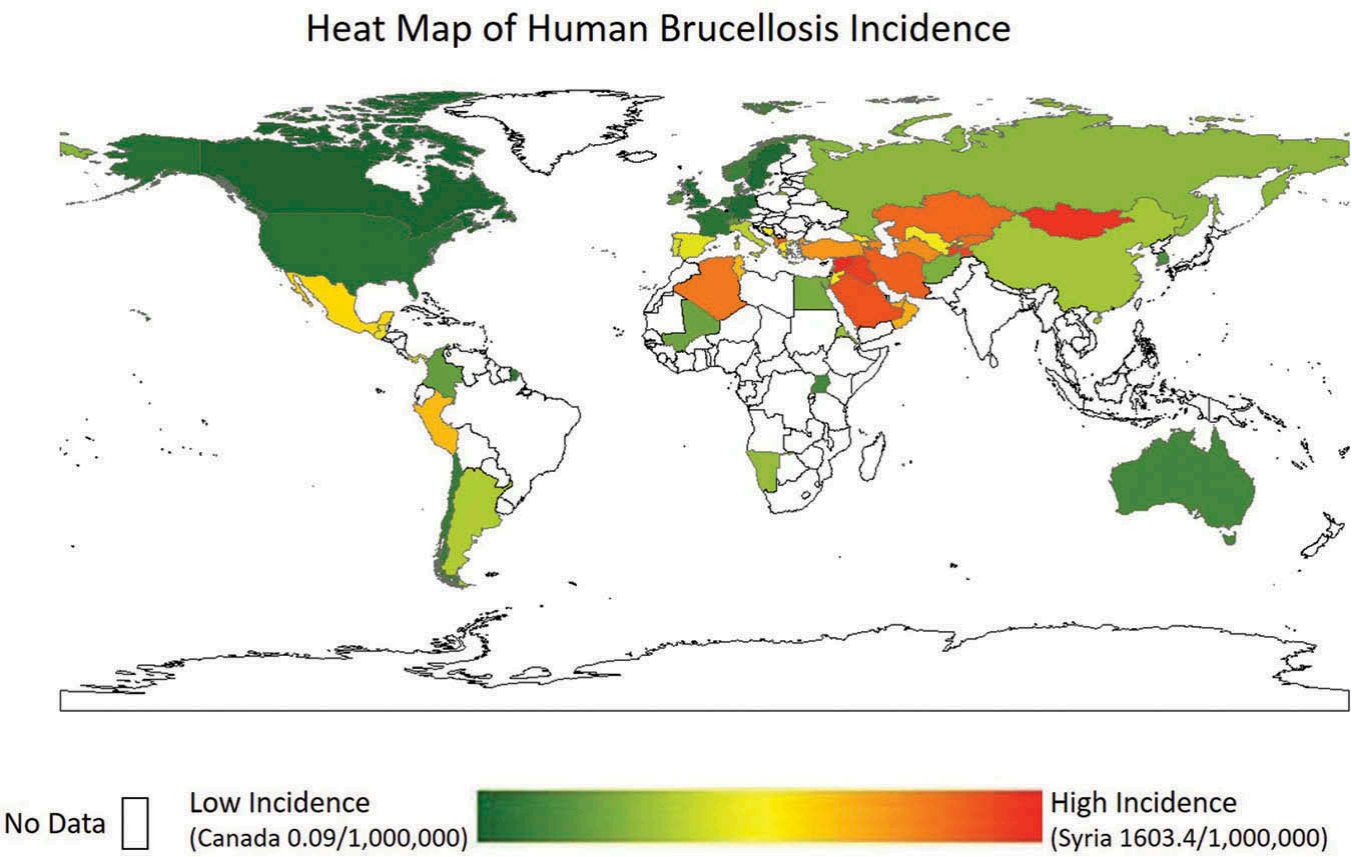
Heat map of human incidence (per 1,000,000 individuals). White space indicates no data. Adapted from Pappas et al., 2006 and other sources [23,27,28].

Although the most widespread zoonosis, brucellosis is classified as a ‘rare disease’ by USA (U.S.) National Institutes of Health. This denotation is applied to most developed countries where incidence is low (USA: 0.40 cases per 1,000,000) [23]. Currently, the U.S. typically sees less than 100 reported cases per year, with most occurring in the south and southwest from illegally imported soft cheeses (unpasteurized) from Mexico [24]. However, in the U.S. true incidence has been estimated at five to 12 times greater, purely from foodborne illness [25]. Syria has been reported to have the highest incidence (1,603.4 cases per 1,000,000 individuals) of any country that report statistics to the WHO [26]. This is followed by Mongolia (3910), Iraq (268.8), Tajikistan (211.9), Saudi Arabia (149.5), and Iran (141.6) [23,26–28]. Several countries have had incidence above 200 in the past decade, but have since decreased dramatically, like Turkey (49.5) and Kyrgyzstan (88.0) [23]. A heat map of incidences is provided in Figure 1. Of note, many countries known to be endemic with human brucellosis are reported as ‘no data.’ This is due to the lack of surveillance and reporting to the WHO as well as the lack of peer-reviewed publications elucidating the incidence of disease. Conversely, the European Union has granted brucellosis-free status to many countries and human cases of brucellosis may have been travel-acquired and thus over-represent the national incidences of disease [26].

**Figure 2.**
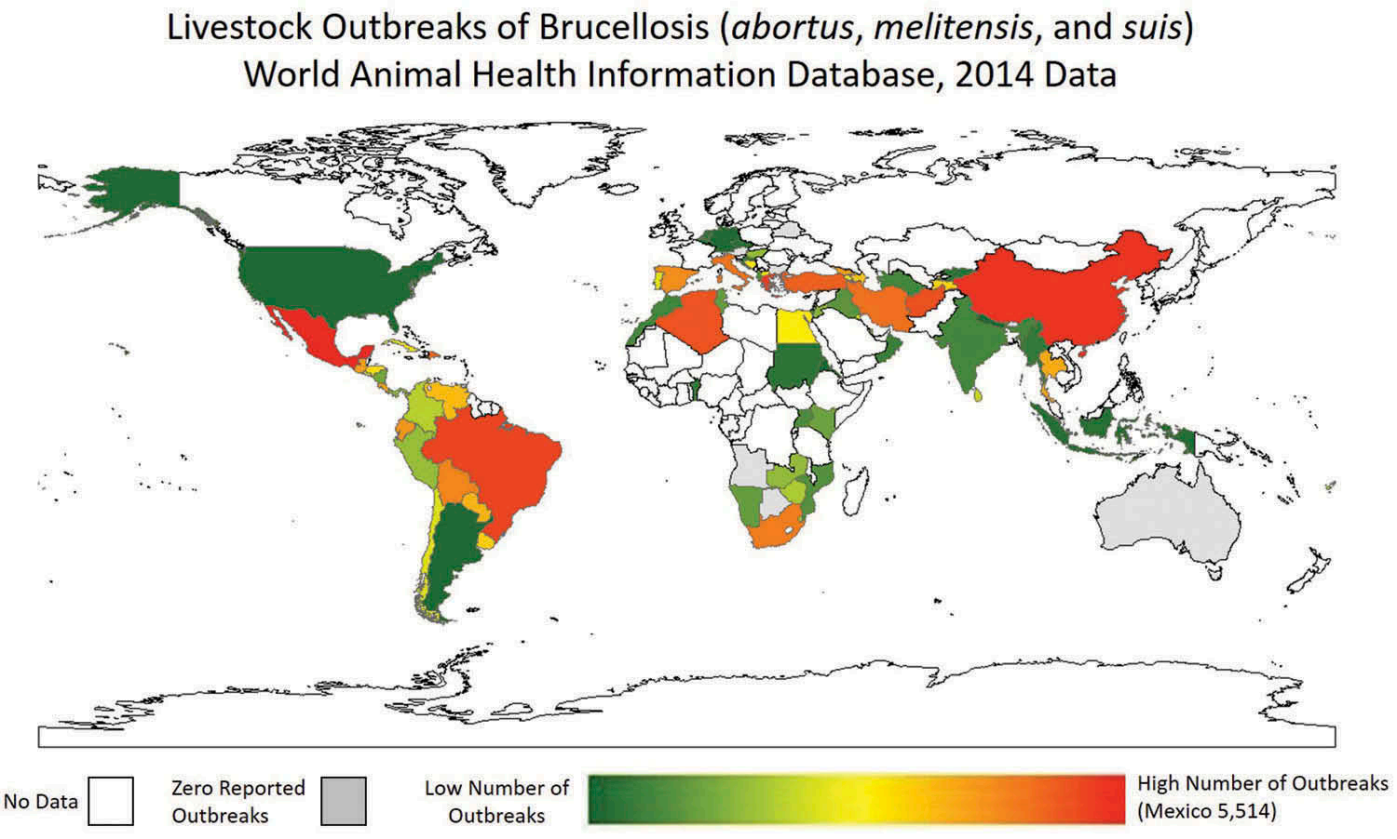
Heat map of number of brucellosis outbreaks (*B. abortus, B. melitensis*, and *B. suis*) in livestock as reported to WAHIS for the last complete year of data, 2014. White space indicates no data. Grey space indicates zero reported outbreaks.

Utilizing the World Animal Health Information Database Interface (WAHIS; http://www.oie.int/wahis_2/public/wahid.php/Wahidhome/Home) a datasheet was compiled to evaluate the number of animal brucellosis cases for *B. abortus, B. melitensis*, and *B. suis*. This interface has the drawback of voluntary reporting into the World Animal Health Organization (OIE) and therefore, suffers from reporting bias. Furthermore, those countries with the financial resources for surveillance are over-represented in this dataset. However, it is the only complete database to compile these statistics. For the calendar year of 2014 (the most recent year with complete information), Mexico had a total number of 5,514 reported outbreak events of brucellosis, 5,174 from *B. abortus*, 340 from *B. melitensis*, and zero from *B. suis*. Other significant countries were China 2,138 (2126, 0, 12), Greece 1,268 (269, 999, 0), and Brazil 1,142 (1142, 0, 0). A heat map of number of reported outbreaks in livestock from *B. abortus, B. melitensis*, and *B. suis* is provided in Figure 2.

## Pathogenesis, clinical presentation, and risk factor in humans

Brucellae can gain entry into the human host via inhalation, ingestion, contact with mucosa, or puncture wounds such as needle sticks [29]. This is followed by an incubation of 10–21 days (but as long as 12 months), a brief bacteremia, and localization to the mononuclear phagocyte system [30]. The parasitic intracellular niche of *Brucella* helps to limit the exposure to the host immune (innate and adaptive) responses and provide protection from antimicrobials [31]. There are two forms of brucellosis; acute and chronic. Untreated, infections can result in undulating fevers due to re-current bacteremic episodes which is followed by new foci of infection (spine, joins, nerve, etc.). Humans, typically, do not produce clinical abortions due to brucellosis infections, thus constituting a dead-end host [29]. Abortions are a primary driver of transmission in animal populations [9].

Differences between human and wildlife/livestock clinical presentation are vast and there are significant differences in diagnostics and treatment strategies for the disease between species. Since humans are able to report symptoms, human brucellosis typically presents with arthralgia, pyrexia (undulant fever), and fatigue. In a retrospective analysis of 1,028 patients over a 10-year period in Turkey, it was found that gender differences were minimal as to the number of cases (female 52.4% vs. male 47.6%) where the mean age of cases was 33.7 ± 16.34 years [32]. Almost 70% of cases were between the ages of 13–44. Arthralgia was the most common reported symptom (73.7% of cases) followed by pyrexia (72.2%), fatigue (71.2%), hyperhidrosis (64.8%), and inappetence (49%). Clinical signs that were significant upon examination were pyrexia (28.8% of cases), hepatomegaly (20.6%), splenomegaly (14.5%), peripheral arthritis (14.3%), and hepatosplenomegaly (10.3%). Laboratory findings often included erythrocyte sedimentation rate elevation > 20 mm/h (59.9%), C-reactive protein positive (58.4%), anemia (40.3%), and transaminase elevation as defined by alanine and aspartate aminotransferase ≥ 50 IU/L (24.8%).

Human case-fatality proportion is very low; < 1% of clinical cases [33,34]. In one study, only five deaths out of 1,028 cases were reported [32]. The major predictor of death was the development of endocarditis. Incidence of endocarditis is around 2% of clinical cases, but responsible for 80% of the fatalities for brucellosis [23]. Cases typically present with chest tightness and shortness of breath combined with fever and fatigue [35]. In a cohort of 10 brucellosis-related endocarditis cases in China, six patients opted for valve replacement surgery and long-term anticoagulation drug regimens. These six cases were followed up for two to three years with a good prognosis. Four patients did not undergo valvular replacement and succumbed to their cardiac-related injuries within one-year of diagnosis [35]. All patients underwent antimicrobial therapies as recommended by the WHO [9]. However, difficulty arises with antimicrobial therapy for infective endocarditis patients in maintaining bactericidal concentrations of antibiotics at the site of bacterial colonization. Furthermore, due to non-descript clinical presentation of brucellosis, diagnosis may be delayed which will provide the bacterium sufficient time to progress to valve damage in these patients. Thus, the recommendation for these patients is continued antimicrobial therapy with replacement of the damaged valves [9]. Consideration should be given to length of therapy in these patients, as extended antimicrobial therapy may be warranted.

Another significant, albeit rare complication of brucellosis is neurobrucellosis. Intracellular invasion of the central nervous system occurs in about 5% of human clinical brucellosis cases [9]. The result of this invasion can be the development of meningitis, meningoencephalitis, brain or epidural abscesses, and/or demyelination disorders [36]. However, even with clinical neurobrucellosis patients, bacterial culture of the cerebral spinal fluid typically results in no growth of the organism [37]. A secondary complication of neurobrucellosis is ophthalmic brucellosis by affecting the optic nerve, either by inflammation or flow change of the optic nerve due to axonal degeneration [38]. Pathogenesis of neurobrucellosis is not well characterized. The vast majority of publications relate to case reports and case series [39].

The majority of clinical complications are osteoarthricular and can occur in 40% of clinical brucellosis cases [9]. The most common osteoarthricular malady reported is peripheral arthritis, typically affecting a single joint [9,40]. Only, 9% of peripheral arthritis cases are found bilaterally or are considered polyarthritis [40]. The second most common osteoarthricular complication is sacroilitis with secondary sciatica [9,41]. Other maladies include spondylitis, peripheral arthritis, bursitis, tenosynovitis, and rarely osteomyelitis. It is more likely to see radiologic evidence and complaints originating from the lumbar vertebrae rather than thoracic or cervical vertebrae [42]. Not surprisingly, those presenting with osteoarthricular brucellosis are more likely to have an elevated erythrocyte sedimentation rate than those without osteoarthricular brucellosis. However, there appears to be no statistical difference between groups with regard to C-reactive protein [40]. Therapeutic failure is three-times higher in osteoarthricular brucellosis compared to brucellosis cases without osteoarthricular complaints [40,43,44].

Genitourinary complications are seen in both humans and animals. In males, orchitis and epididymitis are most frequently reported and account for 6–8% of complications reported [9,32,45]. In females, pelvic abscesses and salpingitis are reported, albeit rarely [46]. However, in human populations there appears to be increased risk of fetal death in women with concurrent brucellosis infections. This association is disputed in peer-reviewed literature [47,48]. However, multiple studies hold that there is a true association between brucellosis infection and spontaneous abortions and fetal deaths [47,49–52]. This association could be explained by maternal toxemia, disseminated intravascular coagulation, or simply bacteremia. In one such study, a group of *Brucella*-seropositive pregnant women were matched against seronegative pregnant women. Spontaneous abortion and fetal death was statistically associated with seropositivity. However, there was no increased risk for preterm labor in brucellosis-infected mothers [49,51].

Risk factors for human brucellosis are limited to consumption of unpasteurized dairy products and occupational exposure. In one such study in Iran, consumers of unpasteurized dairy products had a 3.7 increased odds (95% CI 1.64–8.3) of developing brucellosis compared to controls [53]. Interestingly, this risk for transmission can be decreased (OR: 0.44; 95% CI 0.23–0.85) if individuals are aware of the risk. In a study from Tanzania, occupational risk factors include being an abattoir worker (OR: 7.87; 95% CI 1.42–57.25), presence on the slaughter floor (OR: 5.74; 95% CI 1.25–25.22), and cleaning of the facility (OR: 7.10; 95% CI 1.51–32.05) [54]. In endemic areas of the U.S., high risk occupational groups are National Park Service employees (Prevalence Ratio 3.9; 95% CI 1.50–7.27) and veterinarians (PR 2.5; 95% CI 1.30–4.68) [55];. Use of vaccines, specifically *B. abortus* Strain 19 (S19) had a statistically significant association with anti-*Brucella* antibodies in this sero-survey (Prevalence Ratio 2.7; 95% CI 1.4–5.2).

## Pathogenesis, and clinical presentation in animals

Animal brucellosis infection can occur via multiple differing routes. The most common is via the gastrointestinal tract, but conjunctiva or inhalation are possible [22]. Then, bacterium can translocate to lymphatic vessels and gain access to the circulatory system and cause bacteremia. Tissue tropism includes pregnant uteri, male genital organs, mammary glands, and associated supramammary lymph nodes [22].

Different from human brucellosis, spontaneous abortion in infected ruminants is the hallmark of infection [56]. One of the contrasting differences between species is the presence of the carbohydrate erythritol, which plays a significant role in this clinical presentation in animals [57]. Erythritol is produced by placental tissue of species-specific pregnant animals and can be utilized by brucellae as a growth-stimulatory factor and carbon source, and is preferred over glucose [58]. Release of erythritol from the placenta into the circulatory system causes translocation of brucellae out of lymph nodes and migration to reproductive tissues. The new focus of infection is invasion of the chorionic villi, extending into the cotyledons on the fetal side of the placenta [59,60]. There, bacterium can replicate to a very high level (10^13^ bacteria/gram of tissue) and induce infiltration of inflammatory cells, necrosis of trophoblast, and lead to vasculitis [61,62]. This ultimately leads to compromised fetal-maternal metabolic exchanges, resulting in fetal loss [59]. Fetal and placental tissues and associated fluids expelled in abortion events are the main transmission in animal populations [56]. The bacterium can reside in the environment up to a year, depending on the favorability of conditions (humidity, soil composition, temperature, ultra violet exposure, etc.) [63]. However, presence of scavengers can reduce the time of brucellae in the environment [64,65]. Of note, it has not been recognized that scavengers increase the risk of transmission to livestock, and it is generally believed that scavengers reduce risk of transmission [65]. Should an abortion event not take place, vertical transmission to offspring is still possible to perpetuate the infection. Mammary glands are a target organ for brucellae and secretion of viable bacterial cells through colostrum or milk is another important route of infection. This route is critical in human infections with the consumption of unpasteurized dairy products from infected animals.

The clinical presentation in animal populations largely varies depending on host species. Overall, in bovine brucellosis (*B. abortus*), caprine brucellosis (*B. melitensis*), and swine brucellosis (*B. suis*) animals can present with pyrexia (undulant fever), mastitis, weak offspring, spontaneous abortion, and carpal hygromas [66,67]. Spontaneous abortion is recognized as the cardinal sign of brucellosis infection. The bovine gestational period is approximately nine months. Typically, in bovine brucellosis, fetuses are aborted between the fifth and eighth month of gestation [18,67–69]. Infected pregnant cows or heifers will typically abort once, however, a subset will abort with future parturitions or birth weak calves [70]. That cattle typically only abort in the first pregnancy post-exposure is thought to be explained by acquired immunity after their first abortion event [63,67]. Mastitis is an important feature of the disease. Mammary glands as well as accessory lymph nodes are common niches for brucellae to replicate and evade immune defenses. *Brucella* bacteria are shed in the milk of infected, lactating cows. This is a secondary transmission route to naïve calves; however, it is the most important zoonotic transmission route to humans [12,26]. Infected bulls are thought to be a low risk of transmission to females, mainly due to the inhospitable environment of the vaginal tract [63,71]. Thus, while males can present with epididymis, orchitis, ampullitis, and seminal vesiculitis, it is believed that infected sperm in natural servicing is not a sufficient route of transmission. However, there is an appreciable risk in bull semen that is utilized in artificial insemination due to the intrauterine placement of the semen compared to vaginal deposition during natural servicing [72–74] . Semen from sero-positive bulls has been found to contain *B. melitensis* [75]. In an experimental study of mature bulls, inoculation with *B. abortus* vaccine strain 19 led to the persistent shedding of the this bacteria in semen [76].

Swine brucellosis (*B. suis*) has the most wide-ranging clinical signs and is dependent on age, sex, exposure, and organ involvement [77]. Swine can present with abortion, birth of weak piglets, orchitis, epididymitis, infertility, arthritis, and lameness [67]. Pyrexia in swine is rare and not appreciated in the vast majority of cases. Unlike in cattle, sexual transmission of *B. suis* is the main source of transmission and can induce spontaneous abortions early in gestation [67]. Boars can present with appreciable genital infections, with unilateral testicular enlargement, which can result in infertility [67].

In small ruminants (sheep and goats), clinical signs of *B. melitensis* include abortion and weak offspring [9]. As with cattle, it is thought that abortion typically happens with the first gestation post-infection before acquired immunity can reduce the risk for future abortion events. However, there is still the possibility of future abortion events after the first parturition. Interestingly, in future pregnancies, infectious materials can be shed up to three months post-partum. In male sheep and goats, genital organs are the site of infection and can produce localized inflammation. This can lead to sexual transmission to naïve females in the flock.

*Brucella canis* infections in wild and domestic dogs have potential for zoonotic infections. Human infection with *B. canis* is usually asymptomatic or mild [67]. In canids, clinical signs are late-term abortion, mild pyrexia, and weak-litters. In male dogs, infection of the genital tract can result in epididymitis, orchitis, and prostatitis. Canids are able to clear the infection within two to three years [67]. Diagnostics of canine brucellosis are lacking due to the phenotypic difference of *B. canis* [78].

## Control strategies

Control and eradication strategies vary between developed and developing countries. However, the burden of brucellosis infection is greatest in developing countries. Inconsistent infrastructure (animal health and pasteurization in particular) and lack of funding perpetuate the uncontrolled spread of disease [29]. In developed countries, like the U.S., great strides have been made since the initiation of and provision of funding for control and eradication efforts.

The greatest public health measure to impact zoonotic infections of brucellosis lies with pasteurization of dairy products. The pasteurization process kills microorganisms, like *Brucella spp*., that can potentially cause disease. However, even in developed countries (such as the vast majority of European countries, including those where brucellosis has not yet been eradicated in livestock populations) there is little to no restriction of raw milk and its products [79]. In fact, in the U.S., there is a raw milk movement and several states have passed legislation that allows the partially restricted sale of raw milk and raw milk products to consumers, mainly through a process of partial ownership in a communal animal. Recently, human cases of brucellosis were tied back to a raw milk dairy and implicated RB51 vaccine strain [80]. Many developing countries lack infrastructure to pasteurize dairy products prior to arriving to consumers [81]. In Tanzania, front-end cost of pasteurization facilities is not achievable at the current time and other control strategies are considered more economically feasible. In one study of 59 milk samples in Tanzania, 56% were culture positive for brucellosis [82].

A study conducted in Uganda was able to model a 47% decreased risk of human brucellosis if pasteurization could be implemented in the milk production chain [83]. Effective control programs in developing nations have a benefit to livestock, wildlife, and human populations. Finding a mechanism for funding via international aid as well as buy-in from public and private sectors would bear the best results in control and eradication [84].

## Vaccines

Concentrating control and eradication resources on livestock populations to control infections is typically accepted as the best method to manage brucellosis [12,84]. This can be achieved in one of several ways: vaccination, culling of infected animals, surveillance testing, or a combination of any of these. There is no vaccine that has been developed and approved for use in humans against brucellosis. However, in animal populations there are three main vaccines used for control. RB51 and S19 are directed at *B. abortus* infections in bovids, while Rev1 is used for *B. melitensis* in small ruminants [85]. While these vaccines do not prevent colonization and infection of animals, it decreases the likelihood of an abortion event, which in turn breaks the cycle of transmission and protects the remaining animals in the herd [84].

Vaccination is generally accepted as the most economically favorable measure for the control of animal brucellosis in endemic regions [86,87]. It is important to note that there are two colony morphologies to brucellae that provide background into brucellosis vaccines. One form is a smooth species that contains the smooth O-sidechain lipopolysaccharide (sLPS) [88]. Examples of smooth brucellae are *B. melitensis, B. abortus*, and *B. suis*. These outer membrane domains are recognized as the antigen by serologic assays. The second colony morphology of brucellae is the rough species that are deficient of the O-sidechain lipopolysaccharide (rLPS). Examples of rough brucellae are *B. canis* and *B. ovis*. Currently, there are two vaccines licensed for use in animal populations for *B. abortus*, one is a sLPS (S19), and the second is a rLPS (RB51), that is a smooth-strain mutant that lacks the O-sidechain lipopolysaccharide. A third vaccine, not currently licensed in the US, is a rLPS (S45/20). For *B. melitensis*, there is one vaccine that is licenses for use and is a sLPS (Rev1).

## Culling

In addition to vaccination, culling of suspect or reactor animals based on serology is used in most developed countries. The crux of this strategy relies on testing of herds to determine their sero-status. In developed countries, animals that test in the suspect or reactor range are removed from the herd and either sent to slaughter or are culled by regulatory officials for further definitive testing, such as bacterial culture. Many developing countries do not employ testing at the farm or in slaughterhouses to determine the status of the animals. This is primarily due to the lack of animal health infrastructure.

## Diagnostics

The primary class of diagnostics used in brucellosis surveillance is serologic testing in both humans and animals. There are various serologic tests that are based on the detection of either whole-cell antigen or the sLPS [89]. Overall, serologic tests are an ideal first line test. One major drawback are organisms that share the sLPS (*Yersinia enterocoloitica, Vibrio cholerae, Ochrobactrum anthropi, Salmonella enterica serotype Urbana, Franisella tularensis*, and *Escherichia coli O157:H7*) and cross-react on these tests [90–92]. As with all serologic assays, presence of antibodies indicates exposure, but not necessarily present infection. The inception of serologic assays for brucellosis was in 1897 [93]. Since then assays have been improved and are currently offered in three general classes: agglutination tests; complement fixation tests; and primary binding assays.

Agglutination tests involve the addition of sample serum of unknown status to *Brucella* antigen and observing a pattern of agglutination in either a tube, microwell plates, or paper cards. Some current tests used in animal populations include the standard tube agglutination test (STA), acidified antigen (Rose-Bengal [RBT] or buffered antigen plate agglutination), 2-mercaptoethanol, rivanol (RIV), and the milk ring test. Depending on the assay, they can be relatively easy to perform (STA) or more labor intensive (RIV). Sensitivities are variable (as low as 21% in RBT), however, specificities are usually quite high (96.8–99.3%) [94–96].

Complement fixation tests (CFT) are typically used as a confirmatory test, which the USA Department of Agriculture (USDA) uses as a confirmatory assay on bovine samples. It relies on the presence of the IgG_1_ isotype, which in turn will activate the complement cascade and lysis of an indicator (sheep red blood cells) will not take place. However, this assay is technically challenging and requires multiple reagents to complete, making adoption difficult in developing countries [89]. CFT requires subjectivity in reading test result. Nevertheless, the OIE has recommended this assay for use in international trade [89].

In addition to primary screening agglutination tests, primary binding assays like the fluorescence polarization assay are utilized in series or parallel in the U.S. Serum is used to measure the kinetics (spin) of molecules in solution. An unbound antigen that has a fluorescence marker will spin at a greater speed than an antibody-bound-antigen [97]. This measurement is taken in consideration with background emittance and produces a millipolarization value that can be used to classify an animal as negative, suspect, or reactor for brucellosis.

It is worth noting that replacement of serology is unlikely in the near future. Material cost is pennies per sample (USD), can be performed in minimal time, utilizes ante-mortem samples, and can be field deployable. However, in the U.S., an animal that tests positive via the diagnostic algorithm constructed by regulatory officials will need to be sampled post-mortem for definitive testing. This is completed with the gold-standard diagnostic test of culture. Drawbacks of culture are that it typically requires post-mortem samples from animals, can take up to 14-days, is costly (typically in the U.S., $600/animal USD exclusive of personnel costs), and suffers from imperfect sensitivity. In the U.S., only 30–50% of seropositive animals are culturable [98,99]. This leaves the question of the disposition of 50–70% of seropositive animals that are culture-negative. In the U.S., typically, 22–25 biologic samples are taken from serologically defined suspect or reactor animals. These samples are later plated on five media types in triplicate at U.S. federal labs. Additionally, growing of bacterial cultures provides the possibility of exposure and subsequent infection of laboratory personnel. Considering that brucellosis is the most common laboratory acquired infection in the world, risk to laboratory personnel is high. Therefore, care must be exercised in handling these cultures. *Brucella abortus, melitensis*, and *suis* are currently dually listed in the U.S. as Select Agents by the USA Department of Health and Human Services and the USDA [100]. This designation is given to microorganisms and toxins that have the ‘potential to pose a severe threat to public health and safety’ [100]. Therefore, it is not ideal to amplify live organism for diagnostics. In the U.S., upon culture confirmation of clinical samples, all biologic material must be either destroyed by an approved method or has to be moved to a biosafety level three laboratory or repository for future work. Ultimately, there is a lack of a true gold-standard test that is highly specific and sensitive for use in animal populations. Thus, a better diagnostic test is warranted.

There have been many efforts to develop molecular tests for the detection of brucellosis in post-mortem animal samples. Polymerase chain reaction (PCR) tests have proven both highly sensitive as well as highly specific in human clinical settings. Furthermore, PCR diagnostic testing can take a matter of hours from DNA extraction to results [101]. PCR has been used previously as a diagnostic test for animal brucellosis; however, the test has not been widely implemented due to lack of infrastructure and a wide range of sensitivity values [102]. The first *Brucella*-specific PCR was published in 1990 and was not species-specific [103]. It was validated on S19 vaccine isolates and not tested on spiked or field-collected samples. Additionally, it was unable to differentiate between species of *Brucella* and vaccine strains. The primers and amplicons were never published, so further analysis was not possible [104]. The next advancement in *Brucella* PCRs came in 1992 when primers were designed to amplify a region of the 16S rRNA [105]. Unfortunately, it also amplified the closest known relative to *Brucella, Ochrobactrum anthropi*. Again in 1992, a primer set targeting BCSP31 antigenic periplasmic protein of *B. abortus* was targeted [106]. However, it too amplified all *Brucella* species as well as *O. anthropi*. These primers targeting BCSP31 are still used today in diagnostics in the Middle East, Asia, and East Africa [107–110]. However, it does not exclude vaccine strains from detection and was validated only on post-culture colony isolates [111].

Laboratory results in human patients needs to be viewed with consideration of clinical findings, medical history, hematological testing, and radiographic findings. As stated previously, because humans are able to self-report symptoms, a patient will typically seek care upon an extended febrile episode. This is correlated with the bacteremia associated with a brucellosis infection [112]. Therefore, blood cultures are routinely performed in these patients. Availability of technologies, such as the automated continuously monitored blood culture systems, allows for growth of brucellae in a clinical sample. Additionally, bone marrow cultures can result in 15–20% higher yields of brucellae than peripheral blood cultures [113,114]. Cultures from patients with the acute disease can have sensitivities of 50% to 80%; while the chronic form is less likely (< 5%) to produce a culture [45,113,115]. However, a major drawback is that cultures must be incubated for six-weeks before reporting a negative result [116]. Culture maintains near 100% specificity as colonies will not grow if targeted bacteria is not present in the clinical sample.

Serologic assays are commonly utilized in brucellosis diagnostics and surveillance in human populations. Acidified-RBT-agglutination assays can be conducted similarly to animal diagnostics. However, serology can suffer from false-negative results in chronic cases of brucellosis [117]. Test statistics for RBT are 100% sensitivity and 97% specificity using a ≥ 1:1 cut-off value in acute cases of disease [118]. Of note, this cut-off value is not clinically meaningful as any dilution or mishandling of the sample may lead to false-negative result. Serum agglutination tests (SAT) are also used in human brucellosis diagnostics and utilize IgG, IgM, and IgA antibodies. Test statistics for SAT are 87.4% sensitivity and 100% specificity using a cutoff of ≥ 1:160 [118,119]. A smaller version of SAT is the microagglutination test (MAT) that can be performed in microtiter plates. This assay uses smaller volumes of serum and reagents and is appropriate for running multiple samples at the same time. MAT has the same test statistics as the SAT [118]. Both SAT and MAT have the same downfall of the inability of identifying chronic cases of the disease. Therefore, the cornerstone test for human and animal brucellosis is the enzyme-linked immunosorbent assay (ELISA). The popularity of the ELISA assay can be attributed to the standardization of the assay, reagents, and commercial availability. However, test statistics, per the package insert of commercially available assays, are compared against other ELISA assays and not culture. In the peer-reviewed literature, the cELISA, with a cutoff of 1:10 has a sensitivity of 100% and a specificity of 99.7% [120,121].

## Conclusions

Brucellosis is an ancient disease that still plagues the world, particularly developing nations. While it is the most widespread zoonosis and most common laboratory-acquired infection, there are still knowledge gaps and a need for better diagnostics and vaccines to make inroads towards control and eradication. However, over the past two decades, improvements have been made to better understand the various aspects of human and animal brucellosis. Meanwhile, large numbers of wildlife and livestock, especially in the developing world, are naturally infected with this potential bioterrorism agent. Risk factors have been clearly delineated for brucellosis in human populations, but many developing and war-torn regions lack infrastructure and funding to implement strategies to reduce these risk factors. Therefore, in the U.S., it is beneficial to society to tackle this disease at home and abroad, which will most likely increase expected benefits of control strategies. Immense challenges that remain in controlling and eradicating brucellosis are: (1) to develop and validate novel diagnostics to replace culture, ideally as an ante-mortem assay; (2) to develop efficacious vaccines that provide better protection to animal populations and are differentiation of infected from vaccinated animals (DIVA) compliant; and (3) to address the disease in the natural animal reservoirs and dedicate resources to brucellosis management in animals to reduce the incidence in human populations, effectively applying a One Health framework. Ultimately, with a disease this challenging, all stakeholders must be working together instead of against each other. This disease will not be controlled or eradicated without meaningful collaboration between, local, state, federal, private, and public partnerships.
